# Residence-related factors and psychological distress among evacuees after the Fukushima Daiichi nuclear power plant accident: a cross-sectional study

**DOI:** 10.1186/s12888-016-1134-9

**Published:** 2016-11-24

**Authors:** Naoko Horikoshi, Hajime Iwasa, Norito Kawakami, Yuriko Suzuki, Seiji Yasumura

**Affiliations:** 1 Department of Public Health, Fukushima Medical University School of Medicine, 1 Hikarigaoka, Fukushima City, Fukushima 960-1295 Japan; 2 Tokyo Metropolitan Institute of Gerontology, 35-2 Sakae-cho, Itabashi-ku, Tokyo, 173-0015 Japan; 3 Graduate School of Medicine, The University of Tokyo, 7-3-1 Hongo, Bunkyo-ku, Tokyo, 113-8654 Japan; 4 National Institute of Mental Health, National Center of Neurology and Psychiatry, 4-1-1 Ogawahigashi, Kodaira, Tokyo, 187-8553 Japan

**Keywords:** Psychological distress, Community mental health, Disaster, Relocation, Residence

## Abstract

**Background:**

Relocation following a disaster can impact the psychological well-being of evacuees. This study investigated the associations between residence-related factors and psychological distress among evacuees living in temporary housing after the Fukushima Daiichi Nuclear Power Plant accident.

**Methods:**

Data from 525 participants living in temporary housing were collected. Associations between residence-related factors (frequent relocation, dissatisfaction with the residence, and plan to move to permanent housing) and psychological distress were measured. The psychological distress of evacuees was measured using the Japanese version of the 6-item Kessler scale (K6). We used a cut-off score of five to identify cases with psychological distress, the basis of Kessler’s 6 items for psychological distress.

**Results:**

Multivariate logistic regression analysis (*n* = 418) showed that frequent relocation (OR = 2.05, 95% CI: 1.14–3.66, *p* = 0.016) and dissatisfaction with the residence (OR = 2.48, 95% CI: 1.60–3.83, *p* < 0.001) was significantly associated with psychological distress. After stratifying by gender, dissatisfaction with the residence was associated with psychological distress, and a plan to move to permanent housing was significantly associated with psychological distress in women (OR = 1.93, 95% CI: 1.03–3.63, *p* = 0.041).

**Conclusions:**

Frequent relocation and dissatisfaction with the residence were associated with psychological distress among evacuees following the Fukushima nuclear disaster. Evacuees should be provided with comfortable living spaces, and steps should be taken to reduce repeated relocation of evacuees. Thus, particular attention should be paid to women with a plan to move to permanent housing within this context.

## Background

The Great East Japan Earthquake and tsunami that occurred on March 11, 2011, damaged the Fukushima Daiichi Nuclear Power Plant, leading to the Fukushima nuclear disaster. The impact of the earthquake and the subsequent evacuation of residents into unfamiliar environments due to the radiation hazard are expected to cause psychological distress [1, 2]. According to previous studies, a major long-term health issue following the Chernobyl nuclear accident is psychological distress [3].

Disaster-related relocation affects the psychological well-being of evacuees [4, 5]. Erikson [6], through research with those affected by the Buffalo Creek flood, has reported an effect on psychological distress that was likely caused by the loss of social resources (such as the familiar places and human relationships). Previous studies report many instances of psychological issues following the forced evacuation of homes, due to natural or man-made disasters (such as hurricanes [7, 8], earthquakes [9], terrorist attacks [10], and explosion [11]. Uscher-Pones [12] reported that healthcare disruption, social network change, living condition change, psychological stressors, and individual vulnerability are all associated with heath status within the conceptual framework of the health effects of post-disaster relocation.

Since evacuees following the Fukushima nuclear disaster were suddenly forced into long-term evacuation, their stress is likely considerable. Many evacuees continue to feel anxiety regarding their health, and report mental issues caused by the evacuation [13, 14]. Therefore, the originality of this study is best described by the following three points. First, this study is focused on the evacuees of a complex disaster (i.e. earthquake, tsunami, and nuclear power plant accident). In addition to the natural elements, the nuclear disaster described in the current study was unexpected, unprecedented, and likely the worst nuclear accident in history [1, 2]. Within the framework of this unique occurrence, we aimed to investigate an association between residence-related factors and psychological distress in such complex disasters.

Second, residence-related and psychological problems among evacuees after disasters have been found as well, such as being forced to leave home and be relocated [7, 8], dissatisfaction with the relocation residence [15], and housing damage [16, 17]. However, we feel that the number of those that have been relocated, and plan to move to permanent housing within the relocated area in the future, have not been given adequate consideration in previous studies.

Finally, previous research has shown that sex is one of the factors that affects mental health in the relocation after disaster [5, 18], with women exhibiting diminished resiliency. In addition, youth has been stated as having a high impact on psychological problems after disaster, as a result of increased responsibility, such as home rebuilding [19]. Given this information, the current study focuses on specific differences in age and gender groups.

Furthermore, as women [5, 18, 20] and non-elderly [16, 19] are reportedly particularly susceptible and vulnerable to psychological distress, it is also important to clarify any associations between psychological distress and age or gender group.

Currently, the government has begun to build permanent housing, such as disaster-related public housing, in order to ensure a stable living environment for evacuees. Confirmation of any relationship between residence-related factors and psychological distress could contribute to assessing the psychological well-being of evacuees who started to move to such new environments. In addition, recognition of the factors that affect evacuees after a disaster could assist in developing precautionary measures to reduce psychological distress following future disasters.

Therefore, this study addressed frequent relocation, dissatisfaction with the residence, and plan to move to permanent housing as residence-related factors that might impact psychological well-being. This would be performed by clarifying associations between residence-related factors and psychological distress among evacuees living in temporary housing after the Fukushima nuclear disaster.

## Methods

### Participants

**Fig. 1:**
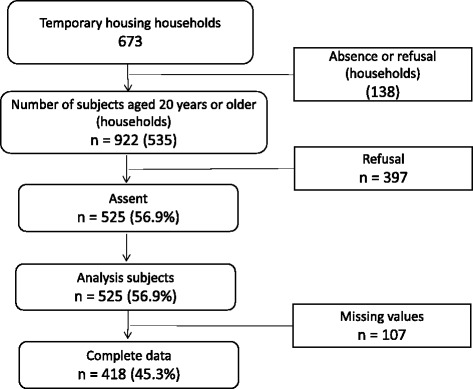
Selection of participants

This study was conducted from October 2013 to January 2014. Subjects were recruited from five temporary housing complexes, located in two municipalities, following the Fukushima nuclear accident. Housing complex registration lists, which included names, head of household, number in family, and ages, were used to initiate contact. Of 673 households, 535 households were successfully contacted. From these households, we selected those who had been living in Fukushima Prefecture before the Fukushima nuclear disaster. These consisted of 922 individuals aged 20 years or older, among whom 525 individuals (response rate: 56.9%) agreed to participate in the study (see Fig. 1).

### Outcome measures

The psychological distress of evacuees was measured using the Japanese version [21] of the 6-item Kessler scale (K6) [22]. The K6 is a self-administered questionnaire consisting of six questions that evaluate depressive moods and anxiety over the preceding 4 weeks on a 5-point scale, ranging from 0 to 4. The total score is an equally weighted sum of the six items, with possible scores ranging from 0 to 24. In this study, we used a cut-off score of five to identify cases with psychological distress, which has been determined as an appropriate threshold for identifying the Diagnostic and Statistical Manual of Mental Disorders, fourth edition (DSM-IV) mood and anxiety disorders in previous studies of community populations in Japan [23, 24].

### Data collection

Data for residence-related factors were as follows: 1) frequent relocation (4 or more times), 2) dissatisfaction with the residence, and 3) plan to move to permanent housing. Dissatisfaction with the residence was scored from 1 (*very satisfied*) to 5 (*very dissatisfied*).

Demographic information was collected via self-administered questionnaire, and included respondent age, gender, employment status, financial circumstances, family size, family members, housing damage, loss of close relatives, and presence/absence of psychiatric disorder. Age was classified into two groups: 20–64 years and 65 years or older. Employment status was classified into three groups: unemployed (which included homemakers), employed, and retired. Financial circumstances were divided into two groups, based on a five-point scale: bad (very poor/poor) and good (average/good/very good). Family members were classified into three groups: living alone, spouses, and other (including family structures such as two-generation households, i.e. elderly parents living with their children). Housing damage was classified into two groups: yes (damage) and no (no damage).

### Statistical analysis

Univariate analysis was used to analyze data from 525 people who completed the self-administered questionnaire and took part in a face-to-face interview. Multivariate logistic regression analysis was used to analyze data from 418 participants with a complete set of data. Also, employment status (unemployed and retired/employed) and family members (living alone and spouses/other) were changed to binary from three values for purposes of analysis.

To examine the association between residence-related factors and psychological distress, we used chi-square tests and Student’s t-tests to address categorical and continuous variables, respectively. For statistical comparisons, chi-square tests followed by residual analysis were applied to employment status and marital status. Subsequently, we used a multivariate logistic regression analysis to assess associations between psychological distress and residence-related factors (frequent relocation, dissatisfaction with the residence, and plans to move to permanent housing). We then repeated the above analysis while stratifying by age and gender groups to examine the interaction. The score was expressed as mean and standard deviation (SD), and *p* < 0.05 was regarded to indicate statistical significance. All statistical procedures were performed using SPSS for Windows (version 21; IBM, Chicago, USA).

## Results

The characteristics of the study population by age (younger people: 20–64 years old; older people: ≥65 years old) and gender groups are shown in Table 1.

**Table 1:** Characteristics of the study population by age and gender

Variable		All participants	<65	≥65	Men	Women
Age	Mean (SD)	66.2 (15.1)	‐‐‐	‐‐‐	66.0 (14.0)	66.3 (15.8)
	<65	199 (37.9)	‐‐‐	‐‐‐	75 (37.5)	124 (38.2)
	≥65	326 (62.1)	‐‐‐	‐‐‐	125 (62.5)	201 (61.8)
Gender	Men	200 (38.1)	75 (37.7)	125 (38.3)	‐‐‐	‐‐‐
	Women	325 (61.9)	124 (62.3)	201 (61.7)	‐‐‐	‐‐‐
Employment status^a^	Unemployed	306 (59.8)	107 (54.9)	199 (62.8)***	78 (40.0)	228 (71.9)***
	Retired	111 (21.7)	15 (7.7)	96 (30.3)	69 (35.4)	42 (13.2)
	Employed	95 (18.6)	73 (37.4)	22 (6.9)	48 (24.6)	47 (14.8)
Financial circumstances	Bad	84 (16.5)	39 (20.2)	45 (14.2)	39 (20.2)	45 (14.2)
	Good	425 (83.5)	154 (79.8)	271 (85.8)	154 (79.8)	271 (85.8)
Family size (mean (SD))	Before disaster	3.7 (2.2)	3.7 (2.2)	3.6 (2.2)	3.5 (2.2)	3.8 (2.2)
	After disaster	2.1 (1.2)	2.5 (1.4)	1.9 (0.9)***	2.0 (1.1)	2.1 (1.2)
Family members^b^﻿	Lliving alone	150 (29.0)	43 (21.7)	107 (33.4)***	59 (29.9)	91 (28.3)
	Spouses	197 (38.0)	56 (28.3)	141 (44.1)	82 (41.6)	115 (35.8)
	Other	171 (33.0)	99 (50.0)	72 (22.5)	56 (28.4)	115 (35.8)
Housing damage	No	174 (34.6)	76 (39.2)	98 (31.7)	61 (31.6)	113 (36.5)
	Yes	329 (65.4)	118 (60.8)	211 (68.3)	132 (68.4)	197 (63.5)
Loss of close relatives	None	308 (61.2)	122 (62.9)	186 (60.2)	118 (61.1)	190 (61.3)
	≥1	195 (38.8)	72 (37.1)	123 (39.8)	75 (38.9)	120 (38.7)
Psychiatric disorder	No	393 (93.6)	145 (91.2)	248 (95.0)	153 (95.0)	240 (92.7)
	Yes	27 (6.4)	14 (8.8)	13 (5.0)	8 (5.0)	19 (7.3)
Frequent relocation	Mean(SD)	4.6 (1.7)	4.6 (1.6)	4.7 (1.7)	4.6 (1.6)	4.7 (1.7)
	<4	119 (23.9)	50 (26.0)	69 (22.6)	48 (25.1)	71 (23.2)
	≥4	378 (76.1)	142 (74.0)	236 (77.4)	143 (74.9)	235 (76.8)
Dissatisfaction with the residence	No	268 (53.7)	108 (56.0)	160 (52.3)	103 (53.6)	165 (53.7)
	Yes	231 (46.3)	85 (44.0)	146 (47.7)	89 (46.4)	142 (46.3)
Plan to move to permanent housing	No	334 (72.1)	119 (67.6)	215 (74.9)	130 (72.6)	204 (71.8)
	Yes	129 (27.9)	57 (32.4)	72 (25.1)	49 (27.4)	80 (28.2)
K6	Mean(SD)	3.6 (4.5)	3.3 (4.3)	3.7 (4.7)	3.0 (4.0)	3.9 (4.8)*
	<5	316 (66.2)	122 (67.8)	194 (65.3)	122 (68.2)	194 (65.1)
	≥5	161 (33.8)	58 (32.2)	103 (34.7)	57 (31.8)	104 (34.9)

Cells show frequencies, with percentages in parentheses. Chi-square tests, Student’s t-test, and Pearson product-moment correlation coefficients were used for analyses. *p < 0.05 ***p < 0.001

Results of statistical analysis using residual analysis

^a^In those aged below 65 years, those who were retired accounted for less than the expected value, while those who were employed comprised more than the expected value. On the other hand, among those aged 65 or older, those who were retired formed more than the expected value, while those who were employed formed less than the expected value. In the case of men, those who were unemployed comprised less than the expected value, and those who were retired or employed formed larger than the expected values. On the other hand, in the case of women, those who were unemployed formed larger than the expected value, and those who were retired or employed formed less than the expected value

^b^In those below 65 years, those who were living alone or spouses formed less than the expected values, while those who chose “other” comprised larger than the expected value. On the other hand, among those aged 65 or older, those who were living alone or spouses formed larger than the expected values; those who chose “other” formed less than the expected value

The sample included 325 women (61.9%), with an average age of 66.2 years (SD = 15.1, range = 21–91 years). The majority of participants were unemployed (*n* = 306, 59.8%), and reported good financial circumstances (*n* = 425, 83.5%). The ratio of employment status differed significantly by age group (*p* < 0.001) and gender (*p* < 0.001); marital status differed significantly (*p* < 0.001) by age group. Family size after the disaster was significantly lower for older people (mean: 1.9 people, SD = 0.9) compared to for younger people (mean: 2.5 people, SD = 1.4; *p* < 0.001), and family size was reduced significantly after the disaster (mean 2.1 people, SD = 1.2) compared to before the disaster (mean 3.7 people, SD = 2.2; *p* < 0.001). In addition, the percentage of those who owned houses before the disaster was 90.6%.

The primary residence-related factor was frequent relocation (*n* = 378, 76.1%); the mean number of relocations was 4.6 (SD = 1.7). Associations between the number of relocation and psychological distress were as follows: ≥3 relocations, *p* = 0.047; ≥4 relocations, *p* = 0.024; and ≥5 relocations, *p* = 0.054. Henceforth, we define “frequent relocation” as four or more relocations. Dissatisfaction with the residence was expressed by 231 people (46.3%), and 129 people (27.9%) planned to move to permanent housing. Risk of psychological distress was present in 161 people (33.8%), but did not differ by age and gender groups. In addition, severe mental illness (K6 ≥ 13) was present in 24 people (4.6%).

Table 2 summarizes the association between residence-related factors and psychological distress. Among the three residence-related factors, frequent relocation (*p* = 0.024) and dissatisfaction with the residence (*p* < 0.001) were statistically significant. In contrast, plan to move to permanent housing was not significantly associated with psychological distress.

**Table 2:** Chi-square tests between residence-related factors and psychological distress

Residence-related factor	Total	K6		*p*
		<5	≥5	
Frequent relocation				
< 4	103 (22.7)	78 (25.8)	25 (16.4)	0.024
≥ 4	351 (77.3)	224 (74.2)	127 (83.6)	
Dissatisfaction with the residence				
No	246 (54.1)	186 (61.4)	60 (39.5)	<0.001
Yes	209 (45.9)	117 (38.6)	92 (60.5)	
Plan to move to permanent housing				
No	308 (72.6)	205 (73.2)	103 (71.5)	0.712
Yes	116 (27.4)	75 (26.8)	41 (28.5)	

Cells show frequencies, with percentages in parentheses

Table 3 shows the results of the multivariate logistic regression analysis, which revealed that psychological distress was significantly associated with frequent relocation (odds ratio [OR] = 2.05, 95% confidence interval [CI]: 1.14–3.66, *p* = 0.016) and dissatisfaction with the residence (OR = 2.48, 95% CI: 1.60–3.83, *p* < 0.001).

**Table 3:** Multivariate logistic regression analysis of the association between participant characteristics and psychological distress

Variable	Total		
	OR	95% CI	*p*
Age	1.01	0.99–1.03	0.287
Gender	1.23	0.78–1.92	0.373
Employment status	1.12	0.59–2.14	0.730
Financial circumstances	0.67	0.37–1.21	0.179
Family members	0.71	0.43–1.18	0.187
Housing damage	0.93	0.83–1.04	0.219
Loss of close relatives	1.21	0.78–1.87	0.390
Frequent relocation (≥4 times)	2.05	1.14–3.66	0.016
Dissatisfaction with the residence	2.48	1.60–3.83	<0.001
Plan to move to permanent housing	1.32	0.81–2.15	0.273

Multivariate logistic regression was used to calculate ORs and 95% confidence intervals (95% CI) after controlling simultaneously for independent variables. *N* = 418

Table 4 shows the results of the above analysis stratified by age and gender groups through the interaction.

**Table 4:** Stratified analysis of the adjusted association between residence-related factors and psychological distress

Variable	Men			Women			<65			≥65		
	OR	95% CI	*p*	OR	95% CI	*p*	OR	95% CI	*p*	OR	95% CI	*p*
Age	1.02	0.99–1.06	0.205	1.01	0.99–1.03	0.504	1.056	1.01–1.10	0.011	1.013	0.97–1.06	0.541
Gender	N/A	N/A	N/A	N/A	N/A	N/A	1.35	0.58–3.14	0.484	1.10	0.62–1.96	0.736
Employment status	1.18	0.45–3.10	0.744	1.16	0.48–2.85	0.741	1.41	0.61–3.28	0.425	0.92	0.28–2.99	0.890
Financial circumstances	0.52	0.21–1.30	0.162	0.82	0.36–1.87	0.634	0.36	0.14–0.93	0.034	1.10	0.48–2.53	0.818
Family members	1.09	0.45–2.62	0.848	0.63	0.33–1.22	0.169	1.22	0.53–2.78	0.643	0.52	0.25–1.05	0.069
Housing damage	0.99	0.81–1.20	0.889	0.91	0.79–1.05	0.206	0.87	0.71–1.07	0.192	0.98	0.84–1.13	0.731
Loss of close relatives	1.30	0.60–2.83	0.502	1.30	0.75–2.24	0.349	1.57	0.72–3.40	0.254	1.08	0.63–1.87	0.779
Frequent relocation (≥4 times)	1.97	0.74–5.26	0.174	1.95	0.93–4.11	0.078	3.06	1.06–8.87	0.039	1.79	0.86–3.75	0.122
Dissatisfaction with the residence	3.55	1.67–7.54	0.001	2.23	1.27–3.91	0.005	3.11	1.36–7.13	0.007	2.43	1.41–4.19	0.001
Plan to move to permanent housing	0.54	0.22–1.33	0.176	1.93	1.03–3.63	0.041	2.47	1.01–6.02	0.047	0.97	0.51–1.83	0.918

Multivariate logistic regression was used to calculate ORs and 95% confidence intervals (95% CI) after controlling simultaneously for independent variables. *N* = 418

An interaction was noted between gender and a plan to move to move to permanent housing. No interaction was found between age and other residence-related factors

With regard to gender, gender and a plan to move to permanent housing was the only statistically significant interaction noted (*p* = 0.011). After stratifying by gender, psychological distress was associated with a plan to move to permanent housing (women: OR = 1.93, *p* = 0.041), and psychological distress was associated with dissatisfaction with the residence (men: OR = 3.55, *p* = 0.001; women: OR = 2.23, *p* = 0.005). With regard to age group, no significant interactions were found. After stratifying by age group, psychological distress was associated with frequent relocation (younger people: OR = 3.06, *p* = 0.039), dissatisfaction with the residence (younger people: OR = 3.11, *p* = 0.007; older people: OR = 2.43, *p* = 0.001), and a plan to move to permanent housing (younger people: OR = 2.47, *p* = 0.047).

## Discussion

We examined the association between residence-related factors and psychological distress in evacuees after the Fukushima nuclear disaster. We found that frequent relocation and dissatisfaction with the residence were associated with psychological distress. We also found that a plan to move to permanent housing was significantly associated with psychological distress in women. In addition, the percentages of participants who scored above the cut-off points of 5 and 13 for the K6 was 33.8% and 4.6%, respectively. The percentages obtained in a previous study were 28.0% and 3.0% [25], respectively. This result suggests that the participants in the present study suffer from higher psychological distress brought about by the Fukushima nuclear disaster.

The influence of relocation on psychological well-being appears strongly within 1 year of relocation, and then weakens over time [26]. However, in this study, there was a significant association between frequent relocation and psychological distress, even approximately 2 years and 7 months after the disaster. Previous studies have found that relocation following disasters increases the risk of psychiatric problems in evacuees [27–29]. Also, Goto [30] found that evacuees who had relocated more than two times after a disaster have an elevated risk of developing psychological problems, and concluded that the relocation itself is a primary stressor. Frequent relocation separates families and changes residential environments, increasing the possibility of psychological distress. Indeed, our study found that family size decreased after the disaster, compared to pre-disaster. Namely, families were forced to live separately following the Fukushima nuclear disaster, because most fathers had to remain in the disaster area due to their jobs, while their wives and children were evacuated to outside the Fukushima prefecture. Because post-disaster mental health issues that result due to the separation of family members often emerge over time, many families will seek support from mental health services [31]. In addition, frequent relocation for evacuees may have changed the social support network, because the temporary housing is located inland, far from their former residences [32], and neighbors in the temporary housing are not the same as before the disaster. Relocation from familiar places and people affect psychological problems [33]. Thus, frequent relocation may increase psychological distress in evacuees following the Fukushima nuclear disaster.

Our findings showed that dissatisfaction with one’s residence was related to psychological distress, consistent with a previous study [15]. The majority of participants lived in privately owned houses before the disaster. Therefore, these individuals may have suffered some distress when situated in temporary housing that was smaller than their own homes before the disaster, and may have felt uncomfortable due to noise from neighbors, such as conversations and other sounds of daily life [34]**.** In addition, their privacy might have been compromised due to more crowded living conditions in temporary housing. Further, it is easy to imagine that the small living spaces might have led to feelings of oppression. Thus, our findings suggest that it is important to evaluate evacuees’ dissatisfaction with their residences in order to understand the predictors of psychological distress for such individuals.

Evacuees might be devoid of hope for their future. A previous study found that lower levels of hope were associated with higher levels of psychological distress [35]. Therefore, we hypothesized that individuals who do not have any plan to move to permanent housing are more likely to have psychological distress. Contrary to expectations, our multivariate logistic regression analysis showed that plan to move to permanent housing was not related to psychological distress. The association might have been counterbalanced and diminished by three possible opposite relationships, which are described below. First, this indicates that plan to move to permanent housing might be a source of stress rather than hope. Indeed, the evacuees have already suffered from stress due to repeated relocation. Second, uncertainty regarding the return to their hometowns might have affected the evacuees. This may be characteristic of the evacuees of a nuclear accident. Third, the results may be explained speculatively by “reverse causality,” in which individuals who had high psychological distress were more likely to have plan to move to permanent housing. Unfortunately, this study could not determine a causal relationship because of the cross-sectional study design.

In another study that was conducted among evacuees of the Great East Japan Earthquake [36], evacuees with no prospects were at higher risk of psychological distress. The reasons why these results differed from the current study are considered the following three points: 1) no evacuees of the nuclear accident were included in the sample, which likely influenced future prospects, 2) individuals that had already settled in permanent housing or were moving into new housing comprised 81% of the sample, which differed from the information obtained from evacuees in the current study (30%), and 3) the findings of the previous study displayed a higher proportion of men and younger people than the current study. As the previous findings differed from the current results, it is necessary for future research to consider additional issues, such as residence patterns, gender, and age, and how these issues might affect plans to move to permanent housing.

In the stratified analysis, dissatisfaction with the residence was found to be associated with psychological distress, regardless of age or gender groups. While no significant interactions were found with dissatisfaction with residence in any particular group, a robust and general result was found across groups. Recent studies have directed attention toward the effects of residential environments or neighborhoods on psychological well-being [15]. With regard to women, psychological distress was associated with a plan to move to permanent housing. As women may have an increased sensitivity to stress hormones, their management of stress is relatively poor compared to men [37], resulting in women being considered to have an enhanced vulnerability to psychological stress. In addition, women tend to be more susceptible to disruption in social ties when compared to men [38]. For this reason, women may be more likely to feel anxious about the loss of social ties that were obtained in a small temporary housing community. Therefore, these results suggest that women who plan to move to permanent housing are prone to experience higher stress. As such, a plan to move to permanent housing was a source of potential psychological distress, rather than a hopeful future prospect for women. Thus, it is necessary to consider that plan to move to permanent housing may lead to anxiety for women.

Conversely, with regard to age group, no interactions were found to be statistically significant. After stratifying, frequent relocation and a plan to move to permanent housing were associated with psychological distress in younger people. Younger people may be more psychologically vulnerable after a disaster because they have responsibilities to society and their family, and a poor capacity for coping with such hardship [11].

The Japanese government enacted the Basic Act on Reconstruction legislation in June 2011, in response to the Great East Japan Earthquake [39]. The purpose of this Act was to promote a smooth and prompt reconstruction following the Great East Japan Earthquake. To provide a secure and stable environment for evacuees, the legislation emphasizes understanding the problems of evacuees, with a focus on the residential environment of temporary housing. Our study suggests that if a disaster evacuation is extensive, the government should provide evacuees with comfortable living spaces that have sufficient space, heating, and soundproofing. Such housing would reduce the risk of repeated relocation of evacuees. In addition, the government should identify high-risk individuals, such as younger people and women who are still in temporary housing 2 years after the disaster, in order to provide more effective and timely support for them.

This study has some limitations. First, there was a lag between the disaster and the interview. Since the prevalence of depression typically decreases as time passes following a disaster [40], the number of people with psychological distress was probably lower than those suffering distress immediately after the disaster. Second, our participants were residing in a particular type of temporary housing. Therefore, it is necessary to study various forms of housing, such as the rental housing provided by the Fukushima prefecture. Third, this study used a cross-sectional design, which does not allow us to draw causal conclusions. Fourth, there might have been a common method bias [41] due to the measured means, because both exposure and outcome were self-reported. Despite these limitations, the present findings provide valuable data to facilitate preparation for future major disasters. Our findings clarify the characteristics of vulnerable people, facilitating the provision of effective support. Further study is required to track longitudinal changes, and to investigate rental housing and have a larger sample of younger participants.

## Conclusions

We found that concern regarding frequent relocation and dissatisfaction with the residence were associated with psychological distress among evacuees following the Fukushima nuclear disaster. Therefore, the government should provide evacuees with comfortable living spaces, and take steps to reduce the risk of repeated relocation of evacuees, so as to reduce psychological distress among this group. The stratified analysis found that plan to move to permanent housing (women) was significantly associated with psychological stress. Plan to move to permanent housing in women may adversely affect mental health rather than provide hope. Thus, women with a plan to move to permanent housing should be paid particular attention.

## Abbreviations

DSM-IV: The Diagnostic and Statistical Manual of Mental Disorders, fourth edition
K6: The 6-item Kessler scale
